# Treating rare diseases with the cinema: Can popular movies enhance public understanding of rare diseases?

**DOI:** 10.1186/s13023-022-02269-x

**Published:** 2022-03-05

**Authors:** Jan Domaradzki

**Affiliations:** Department of Social Sciences and Humanities, Poznan University of Medical Sciences, Rokietnicka 7, St., 60-806 Poznan, Poland

**Keywords:** Rare genetic diseases, Cinema, Popular movies, Science and movies, Science education

## Abstract

**Background:**

Rare diseases (RDs) constitute an important public health issue. However, although public awareness campaigns focus on the improvement of undergraduate and postgraduate education, also popular culture may serve as an educational tool in this field. This study aims to analyse how rare genetic diseases are depicted in popular movies.

**Methods:**

Twenty popular movies on RDs were analysed quantitatively. The main categories included in the coding frame were: disease, patient, physician/scientist and psychosocial issuses related to RDs.

**Results:**

The majority of movies do not contain adequate scientific information on RDs. Consequently, their cinematic image is either inaccurate or simplified. However, the cinema does take up some important topics in the field of RDs and highlight their ethical, psychosocial, legal or economic dimension: the diagnostic and therapeutic odyssey, the role of RD patients’ advocacy groups in the production of scientific knowledge, the problem of orphan drugs, the stigmatisation of and discrimination against RD patients, and the impact of diagnosis on one’s concept of self and parents’ feelings of guilt.

**Conclusion:**

Although popular movies mostly focus on RD patients’ problems of daily living and rarely describe clinical aspects of RDs, they do have an educational potential. Thus, movies can help to raise the public’s awareness on the psychospocial and economic problems faced by RD patients and their families.

## Introduction

Rare diseases (RDs) are chronically debilitating or life-threatening conditions with a high level of complexity despite their low prevalence of less than 5 per 10,000 persons [1]. Although they do seem rare, it is estimated that 6–8% of the world’s population, i.e. approximately 300–350 million people, are affected by RDs. This means that one out of every 15 persons worldwide could be affected, or that if all of the people suffering from RDs lived in one country, it would be the world’s third most populous country. While there are between 6,000 and 8,000 RDs, 80% are caused by genetic mutations and 50% of RDs patients are children. Simultaneously, although in recent decades there has been progress in the research, development and marketing of orphan drugs, due to their genetic origin no cure exists for the vast majority of RDs. Indeed, even though during the last four decades the number of orphan drugs designations granted have more than quadrupled, still it is estimated that only 5% of RDs have approved drug treatment. Moreover, 75% of orphan products approved treat only one RD and have no other use [2, 3]. What is equally problematic is that approximately 50% of RDs do not have a disease specific foundation supporting or researching their condition [4, 5]. Another problem is that RDs are often overlooked by medical education [6–11], government officials and policy makers, public health programs and news media [12, 13]. Indeed, RD patients and their caregivers often complain over the lack of knowledge about RDs from healthcare professionals and feel frustrated with their negative experience with health and social care services and express a general lack of trust in the standard of healthcare [14–16].

Thus, it has been argued that because RDs constitute an important medical and social challenge and an urgent public health issue, they should be prioritized by policymakers, healthcare providers and medical education campaigns alike [17–20]. Although in recent years a lot has been done to enhance RD policies, through orphan drug reimbursement systems and government actions [21–23], there are still many gaps in the public awareness on RDs. Consequently, calls for initiatives to improve both healthcare professionals’ knowledge and the public’s awareness on RDs have emerged [6–11]. Simultaneously, while many focus on the improvement of undergraduate and postgraduate education, it has been suggested that also popular culture, and movies in particular, may serve as an educational tool in this field [24–27]. The reason for this is put forward that while only a small portion of the general public have ever met a patient suffering from an RD, the majority derive their impressions on RDs from other sources, primarily the entertainment media and popular culture. And because during the last decades, parallel to the dynamic progress in genetic knowledge and the development of novel orphan drugs, many movies on RDs have been released, the cinema may familiarize the public with diseases that are typically foreign to the everyday medical practice and help facilitate the transfer of knowledge and attitudes on RDs to medical students, healthcare professionals and the public.

Thus, this study analyses how rare genetic diseases are presented in popular movies. At the same time, while it was primarily focused on the dominant images of RDs in films, I was also interested in the following questions:

- How do films depict RDs? Do they familiarize the audience with the knowledge on the symptoms, etiology and treatment of RDs?
- How do films frame RD patients?
- What images of physicians and scientists emerge from popular movies?
- What are the psychosocial, ethical and economic implications of RDs depicted in movies?

## Material and methods

The sample of movies was designed according to a content-based criteria. A movie search was conducted in May 2021 using the two electronic online movie databases: Filmweb (http://www.filmweb.pl) and Internet Movie Database (http://www.imdb.com). Then the available plot descriptions were compared with predefined key words: “rare disease”, “genetic disease”, “orphan disease”, “terminal illness”. Additionally, data from the movie databases included the year of production, genre, producing country and director.

To provide more homogenous results, television series with a medical theme, i.e. *ER*, *The Good Doctor*, *Code Black*, *Grey’s Anatomy*, *Private Practice* or *The Resident* were excluded. Although these movies could add something into the discussion about the cinematic image of RDs, as some episodes featured patients with such diseases, this research was limited only to feature films where RDs were the main theme. Documentary movies [28] on RDs, such as: *My Flesh and Blood*, *Our curse*, *Life according to Sam*, *Rare* or *Esto no es una persona* were also omitted. While especially this type of movies would add some diversity to the genres, they have been disregarded because what I was rather interested in was how popular movies framed orphan diseases. Movies picturing RDs that are not of genetic origin, such as *Brain on Fire* (anti-NMDA receptor encephalitis) or *Awakenings* (Encephalitis lethargica) were also excluded. Similarly, films where the RD is a backstory and was only mentioned but was not developed, i.e., *The Big Sick* (adult-onset Still’s disease), *Shallow Hal* (spina bifida), *Glass* (osteogenesis imperfecta), *The 6th Day* (cystic fibrosis), *Dancer in the Dark* (Pigmentary retinitis), *The Sea Inside*/*Mar adentro* (cadasil), *Amélie*/*Le Fabuleux destin d’Amélie Poulain* (osteogenesis imperfecta) or *Unbreakable* (*osteogenesis imperfecta*), were also omitted. Also movies addressing fictional RDs, i.e. *Star Trek Nemesis* (“Shalaft’s Syndrome”), *Blade Runner* (“Methuselah Syndrome”), *The Curious Case of Benjamin Button* (“Button disease”) or *Waterless* (undefined allergy to water) were not taken into consideration. Strictly for this reason, although the main character in the movie *Poder* has albinism, it was excluded because he has also paranormal powers, including telepathy. Finally, movies in which parents make their children believe they suffer from an RD (i.e. severe combined immunodeficiency) due to their overprotective nature, were also excluded (*Bubble Boy* and *Everything, Everything*).

Simultaneously, I am aware that while the selection of the movies included in the analysis neither is nor could be representative, it does not exhaust other interpretations. However, I believe that the selected movies possess a narrative utility because they provide a unique insight into the cultural images of RDs in movies that can easily be recognized by the public. I am also convinced that this research shows that movies both reflect and (re)construct the social images of rare genetic disease. Additionally, to make sure that the selected movies reached a wider audience, their box office have been checked (http://www.boxofficemojo.com), which, at least to some degree, reflects the scale of their reception.

**Fig. 1:**
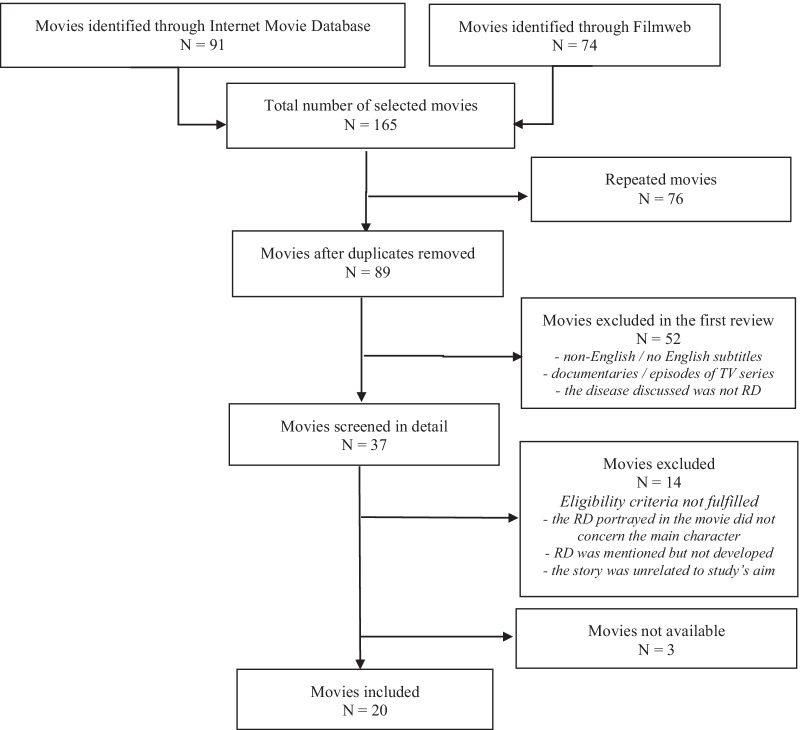
Review and selection process of movies included

The initial search identified 165 movies, which were first assessed based on their titles and plot summaries (Fig. 1).

After removing duplicates, 89 films were evaluated in more detail. The first stage of the screening excluded 52 movies that were non-English movies or had no English subtitles, were documentaries or episodes of television series, or because the disease discussed in the movie was not relevant to the key issue of rare genetic diseases. From the remaining 37 movies, fourteen were excluded because they did not meet the eligibility criteria, i.e. the RD portrayed in the movie did not concern the main character, was only mentioned but not developed or because the story was incongruous to this study’s aim. Additionally, three movies were not available. Thus, of all the movies that met the inclusion criteria, 20 were included, watched and analysed quantitativly (Table 1).

**Table 1:** List of selected movies (N = 20)

Movie tittle	Type of disease	Year	Genre	Country	Director	Box office
*The Boy in the Plastic Bubble* (TV movie)	Severe combined immunodeficiency	1976	Biography, Drama	USA	Randal Kleiser	No data available
*The Elephant Man*	Proteus syndrome^a^	1980	Biography, Drama	USA	David Lynch	$26,023,706
*Mask*	Craniodiaphyseal dysplasia	1985	Biography, Drama	USA	Peter Bogdanovich	$48,230,162
*Crystal Heart* (*Corazón de cristal*) (TV movie)	Severe combined immunodeficiency	1986	Drama, Romance	Spain/USA	Gil Bettman	No data available
*Alex: The Life of a Child* (TV movie)	Cystic fibrosis	1986	Biography, Drama	USA/Canada	Robert Markowitz	No data available
*Lorenzo’s Oil*	Adrenoleukodystrophy	1992	Biography, Drama	USA	George Miller	$7,286,388
*Children of the Dark* (TV movie)	Xeroderma pigmentosum	1994	Drama	USA	Michael Switzer	No data available
*Jack*	Werner syndrome	1996	Comedy, Drama	USA	Francis Ford Coppola	$58,620,973
*Sixth Happiness*	Osteogenesis imperfecta	1997	Drama	UK	Waris Hussein	No data available
*The Mighty*	Morquio syndrome	1998	Comedy, Drama	USA	Peter Chelsom	$2,652,246
*Simon Birch*	Osteogenesis imperfecta	1998	Comedy, Drama	USA	Mark Steven Johnson	$18,253,415
*Jack and Jill vs. the World*	Cystic fibrosis	2008	Drama, Romance	USA/Canada	Vanessa Parise	No data available
*Paa* (*Father*)	Progeria	2009	Comedy, Drama	India	R. Balki	$9,696,629
*Extraordinary Measures*	Pompe disease	2010	Drama	USA	Tom Vaughan	$15,134,293
*And I'll Be Dead Tomorrow Noon* (*Und morgen mittag bin ich tot*)	Cystic fibrosis	2013	Drama	Germany/ Netherlands/Belgium	Sacha Polak	No data available
*Wonder*	Treacher Collins syndrome	2017	Drama, Family	USA/Hong Kong	Stephen Chbosky	$306,209,289
*Midnight Sun*	Xeroderma pigmentosum	2018	Drama, Romance	USA	Scott Speer	$27,365,467
*Five Feet Apart*	Cystic fibrosis	2019	Drama, Romance	USA	Justin Baldoni	$91,527,795
*Ondine*	Congenital central hypoventilation syndrome	2019	Drama	Poland	Tomasz Śliwiński	No data available
*More Beautiful for Having Been Broken*	Fanconi anaemia	2019	Drama	USA	Nicole Conn	No data available

^a^Although for many years it has been argued that Joseph Merrick, the cinematic ‘elephant man’, was afflicted with Neurofibromatosis type I, most recent studies have demonstrated that he actually suffered from Proteus syndrome [29]

A content analysis started with familiarization with the data, which involved watching all the movies. After becoming immersed with their content, a standardized and structured data extraction tool was developed to include the most important features present in the films. The main categories included in the coding frame were:

- **rare disease:** this category refers to the way rare genetic disease is depicted in films and includes: the type of RD, description of its symptoms, etiology, therapy, suffering, type of care, death and dying;
- **RD patient and the family:** this category embodies patients’ demographic characteristics, including, sex, age and ethnicity, prognosis, place and type of death, patient’s self-image and emotional reactions, patient’s problems and needs, family reaction;
- **physician and/or scientist:** this category refers to physician’s demographic characteristics, including, sex, age, ethnicity, specialization, position, type of research, place of work, interest in patient’s disease including physician–patient communication;
- **psychosocial issues related to RDs** which included stigmatization, isolation/social exclusion, discrimination and reduced life opportunities.

These four categories were selected because they describe and identify the key points in the scientific literature [30–33]. Moreover, I believe they represent the public understanding of RDs in movies.

In the last stage of analysis, all the movies were viewed carefully a second time and every scene or passage that supported pre-determined categories mentioned above was noted on the coding sheet. To achieve this I have taken into account both verbal and nonverbal messages. After comparing all notes from each movie, the repetitive patterns were found and analyzed.

## Results

### Rare diseases’ symptoms, etiology and treatment

While the cinema covers a wide variety or RDs, it was cystic fibrosis (n = 20%), severe combined immunodeficiency, osteogenesis imperfecta and xeroderma pigmentosum (n = 10% apiece) that were the most prevalent in the movies (Table 2). Simultaneously, although most of the movies introduced the disease either by mentioning its name (75%), describing the etiology of the disease (50%) or by highlighting its most common symptoms (75%), still many others give only a very brief description of the disease or provide no such information whatsoever. Thus, although films present a variety of symptoms, ranging from immunodeficiency, abnormal growth, loss of weight, breathing difficulties, problems with movement, pain and mental retardation, and highlight the complexity and severity of RDs, frequently, they lack a more detailed description.  Thus, the RD’s explanation is often reduced to a couple of sentences expressed in pseudoscientific jargon and rests on simplifications. For example, while very few movies explain the molecular mechanism of the given disease, many others contain very little scientific information on the RD discussed in the movies. Consequently, because the image of some RDs depicted in movies is often inaccurate or simplified, the educational nature of movies on the clinical dimension of RDs is very limited.

**Table 2:** Characteristics of rare diseases in the movies (N = 20)

	N	(%)
Type of disease		
* Severe combined immunodeficiency*	2	10
* Craniodiaphyseal dysplasia*	1	5
* Proteus syndrome*	1	5
* Adrenoleukodystrophy*	1	5
* Xeroderma pigmentosum*	2	10
* Werner syndrome*	1	5
* Osteogenesis imperfecta*	2	10
* Morquio syndrome*	1	5
* Cystic fibrosis*	4	20
* Progeria*	1	5
* Pompe disease*	1	5
* Treacher Collins syndrome*	1	5
* Fanconi anaemia*	1	5
* Congenital central hypoventilation syndrome*	1	5
Mentions/describes the disease		
Yes	15	75
No	5	25
Describing etiology of disease		
Yes	10	50
No	10	50
Describing/explaining symptoms		
Yes	15	75
No	5	25
Symptoms occurring in movies*		
Immunodeficiency	3	15
Abnormal growth	7	35
Problems with movement	8	40
Breathing difficulties	7	35
Premature aging	2	10
Muscle weakness	4	20
Bone fractures	2	10
Skin problems, i.e. blisters	3	15
Seizures, convulsions	2	10
Metabolic/gastrointestinal problems	3	15
Neurological problems	2	10
Heart problems	4	20
Mental retardation	2	10
Pain	7	35
Caught	6	30
Copious phlegm	4	20
Blood spitting	1	5
Fatigue/tiredness	2	10
Loss of weight/inability to gain weight	6	30
Loss of hair	1	5
Loss of appetite	1	5
Loss of concentration	1	5
Loss of consciousness	2	10
Loss of speech	1	5
Mood swings	1	5
Depression	1	5
Therapy		
Medications	6	30
Operation	3	15
Inhalations	4	20
Enzyme therapy	1	5
Unspecified experimental therapy	4	20
Does not mention	4	20
No therapy	15	20
Medications		
Yes	6	30
No	14	70
Suffering		
Yes	9	45
No	11	55
Hospital/palliative care		
Yes	7	35
No	13	65
Death		
Yes	10	50
No	10	50
Patient Advocacy Group		
Yes	2	10
No	18	90

*Many symptoms appeared in more than one movie

Addtionally, although many movies depicted some kind of treatment, including medications (30%), operations (15%) or some experimental therapy (20%), the majority either do not mention these (20%) or stress explicitly that there is no evaluable treatment for RDs (20%). 70% of the cinematic patients did not receive any type of medciations, 45% suffered pain and 35% received hospital or palliative care. In 50% of the movies the disease leads to the patient’s death. Interestingly, only two movies made any reference to a patient advocacy group (35%).

### Patients and their families

RD patients presented in the movies were predominantly male (60%), Caucasian (80%), or children (56%) who lived in rather stable social environments and were cared for by their families at home (60%) (Table 3). The mortality rate for all twenty five patients depicted in the movies was 44%. Of those who died, 64% passed away at home. Additionally, while 91% of deaths were caused by the disease itself, one character chose physician assisted suicide. Although most of these cinematic patients accepted their disease and the vision of inevitable death (84%), some also experienced such negative emotions as depression (28%), denial (12%) or anger (4%). The most commonly depicted medical obstacles were the lack of available treatment (84%), high cost of drugs and care (40%) and lack of scientific knowledge (24%). 72% of patients’ families showed those suffering the RD their love and support, although some denied the disease (16%), blamed themselves for passing it on to their children (12%) or were indifferent (24%).

**Table 3:** Patient characteristics (N = 25*)

		(%)
Sex		
Male	15	60
Female	10	40
Age		
Child	14	56
Adolescent	7	28
Adult	4	16
Ethnicity		
Caucasian	20	80
Afro-Americans	3	12
Hindu	2	8
Type of care		
Homecare	15	60
Hospital care	4	16
Home and hospital care	6	24
Prognosis		
Survives	14	56
Dies	11	44
At home	7	64
In the hospital	4	36
Type of death		
Natural death	10	91
Physician assisted suicide	1	9
Patient’s reactions		
Denial	3	12
Anger	1	4
Depression	7	28
Acceptance	21	84
Patient’s problems and needs		
Lack of access to correct diagnosis	2	8
Lack of information	3	12
Lack of scientific knowledge	6	24
Lack of appropriate quality healthcare	4	16
Lack of treatment	21	84
High cost of drugs and care	10	10
Inequities in availability of treatment and care	3	12
Disturbance in family dynamics	6	24
Family reaction		
Care/support	18	72
Feeling of blame	3	12
Disinterest/indifference	6	24
Lack of understanding	3	12
Denial	4	16

*Some movies covered more than one patient

### Images of physicians and scientists

The prototypical physician portrayed in movies was also Caucasian (75%), middle aged (46%) and male (83%) (Table 4). While 75% of the cinematic scientists were leaders or chief physicians working in a hospital (92%), 25% were assistants. Significantly, the majority of the latter were females. Additionally, most of the physicians engaged in the caring process were framed as benevolent, altruistic, empathic and committed doctors, caregivers, advisors and counsellors, who struggled to help their vulnerable patients and assisted their families in their distress (75%) as well as fought the bureaucracy that impedes patients’ access to novel treatment options.

**Table 4:** Images of physicians and scientists (N = 24*)

	N	(%)
Sex		
Male	20	83
Female	4	17
Age		
Young	3	12
Middle age	11	46
Older	10	42
Ethnicity		
Caucasian	18	75
Asian	2	8.3
Hindu	4	16.7
Position		
Leader/chief physician/scientist	18	75
Assistant	6	25
Type of researcher		
Physician	22	91.7
Individual researcher	2	8.3
Place of work		
Hospital/university clinic	22	92
Private corporation	1	4
University	1	4
Attitude toward patient		
Care/support	18	75
Disinterest or insensitive behavior	6	25
Physical appearance		
Attractive	11	45.8
Not attractive	13	54.2

*Some movies covered more than one physician

### Psychosocial issues related to RDs

Interestingly, irrespective of the veracity of the description of RDs, the vast majority of films highlighted RDs related psychosocial issues (Table 5). Thus, all patients struggled with negative social consequences of their diseases, such as reduced life opportunities (60%), stigmatisation (48%), isolation/social exclusion (48%) or discrimination (32%). Simultaneously, while concerns regarding the negative impact of the disease on the patient’s quality of life and life opportunities were the most common trope, many films focused on how RD patients and their families are stigmatized because of the genetic nature of the disease. Thus, some films emphasized that RD patients experience structural stigma within societal structures, i.e. healthcare settings, workplaces or educational institutions. Simultaneously, while some films showed that healthcare providers’ lack knowledge and the skills required to manage RDs (i.e. *The Elephant Man*, *Lorenzo’s Oil*), others stressed how employers, co-workers or schools refuse to meet patient’s needs (i.e. *Mask*, *Children of the Dark*, *Wonder*). Other films focused on interpersonal stigma which occurs in patients’ interactions with other individuals, including peers or neighbours. Thus, by showing lack of understanding from others, the movies suggested that RD patients suffer not only from the lack of diagnosis or treatment but also social support (i.e. *Simon Birch*, *Mask*, *The Mighty*). Finally, some films highlighted how RD patients suffer from a felt stigma and how they become somehow accustomed to being subjected to discrimination from others. Thus, *The Boy in the Plastic Bubble*, *Mask*, *The Mighty*, *Children of the Dark*, *Wonder* or *Ondine* show how RD patients, especially those with visible symptoms, feel shame about how they look or act because of their RD. All in all, most movies under the study showed how discrimination resulting from genetic condition reduces one’s life opportunities, contributes to social inequity and negatively affects those with the stigmatized trait.

**Table 5:** Psychosocial issues related to RDs

	N	(%)
Stigmatisation	12	48
Isolation/social exclusion	12	48
Discrimination	8	32
Reduced life opportunities	15	60

*Some movies covered more than one ELSI

## Discussion

Although cinematic depictions of more common diseases have been the object of previous studies [34–37], to the best of my knowledge, this is the first descriptive analysis of the images of rare genetic disease in popular movies. Even though, compared to the total number of movies or those dealing with more common diseases, only a small number of films focus on rare genetic diseases,^1^ it must be acknowledged that over time this number is slowly increasing. Simultaneously, the vast majority of analysed movies were either produced in the United States or in cooperation between the US and European or Asian countries. However, this should not surprise, as the American film industry has a dominating power on the global movie market and its impact on popular culture and the global audiences is distinctive [38, 39].

At the same time, it should be stressed that because the majority of movies do not explain the specificity of RDs and often lack a more detailed scientific information on the RDs discussed in the movies, their cinematic image is rather superficial and vague. Cosnequently, the educational role of movies on the clinical dimension of RDs is limited. However, this should not surprise, as the main aim of popular culture is to entertain the audience rather than to educate. Because film is also a commercial product which needs to find an audience in order to make a profit, most popular movies introduce RDs only by highlighting their symptoms without adding a more detailed description. Conseqeuntly, they often rest on simplifications and reduce the information about the genetic aspects of RDs to a minimum in favour of making the picture more attractive and dramatic. Moreover, rarely do they explain the genetic basis of the disease discussed in the movie, and the majority do not contain any scientific information on RDs. Finally, some RDs are presented inaccurately and/or incorrectly. Especially the older movies, such as *The Boy in the Plastic Bubble*, *Mask* or *Jack* provide the audience only with the most basic information about the RD discussed in the movie, such as its name, that it is rare, and of genetic origin. On the other hand, while the cinematic description of cystic fibrosis in *Jack and Jill vs. the World* or *And I’ll Be Dead Tomorrow Noon* or the Treacher Collins syndrome in *Wonder* is reduced to a couple of sentences expressed in scientific jargon, other movies, i.e. *The Elephant Man*, *Crystal Heart* or *Simon Birch* give no such information whatsoever (sometimes they even do not mention the name of the disease). What is also problematic is that many movies use RDs solely as a Hollywood teen love plot device. Thus, as they focus more on patients’ desire for the mundane pleasures in life [27] they romanticize the terminal character of the RD and somehow trivialize it (*The Boy in the Plastic Bubble*, *Crystal Heart*, *Jack and Jill vs. the World*, *Midnight Sun* or *Five Feet Apart*). Finally, while some movie explore caregivers hopes and struggle to find a test or drug, they often falsely paint a picture of a miracle cure (*Lorenzo’s Oil*).

However, this is not surprising because numerous studies indicate that cinematic depictions of common diseases too are often stereotypical, inaccurate and characterized by misinformation about symptoms, causes, and treatment. For example, people suffering from schizophrenia are frequently portrayed as unpredictable, violent, dangerous or committing homicide. Moreover, while the filmmakers tend to focus on hallucinations, traumatic events and violence, schizophrenic patients’ socioeconomic status is also unrealistic [36, 40–42]. The cinematic image of autism spectrum disorder is also very far from being an accurate, representative or useful one—the reason for this being that films often concentrate on the extreme features of autism and reinforce the negative stereotypes of persons with ASD either as ‘freaks’ or ‘geniuses’ who speak in a monotone or rhythmic manner and have all the expected tics. Simultaneously, high functioning forms of autism are given prominence [43, 44]. While there is a progression in the understanding of epilepsy in many movies, it continues to be associated with the supernatural. Thus, although its older associations with insanity, uncontrolled violence or victimization tend to normalize, cinematic depictions of epilepsy still refer to demonic or divine possession, genius, lunacy, delinquency and “otherness” [45, 46]. Finally, films related to cancer focus on uncommon cancers such as leukemia and brain tumors, while such common types of cancer as breast cancer are barely represented. Consequently, films portray cancer patient’s chances of survival inaccurately and in spite of the progress of cancer treatments they reinforce the stereotype of cancer as an incurable and lethal disease. Thus, it is suggested that popular images of cancer can instill carcinophobia, especially in that cinematic cancer often does not match the epidemiological data as filmmakers prefer younger patients and those from the higher social classes [47–49].

Nevertheless, some movies dealing with RDs contain “kernels of scientific truth” [27, 50]. In particular, *Lorenzo’s Oil*, *The Mighty*, *Extraordinary Measures* and *Five Feet Apart*, provide the audience with detailed scientific information about the diagnosis of the RD, its aetiology, signs and symptoms, the availability of genetic testing and management, including therapy and medications or recent breakthroughs in understanding the disease. Consequently, the painful realities of adrenoleukodystrophy, Morquio syndrome, Pompe disease and cystic fibrosis depicted in these movies are particulary reliable and may increase public knowledge on these diseases.

This study also confirms previous findings regarding the cultural representations of scientists which show that although especially in earlier movies there was a tendency of the vilification of scientists and the good scientists were in the minority, from the 1990s and 2000s onwards the cinema has seen the ascendance of heroic scientists, who are pictured as idealist and hardworking professionals. Thus, while Andrew Tudor’s [51], Roslynn Haynes [52, 53] and Sevan Terzian and Andrew Grunzke [54] showed that cinematic scientists are mainly framed either as foolish scientist-inventors or dangerous and deluded madmen, this research confirms observations from other research that suggest that in the twenty first century images of medical scientists are mainly positive. Thus, although still scientists are perceived in highly stereotyped, often unfavorable, ways, increasingly, they are framed as heroes [55–57]. Indeed, this research shows that in movies dealing with RDs physicians/scientists are mainly portrayed either as brilliant researchers who struggle to find a cure or as altruistic and empathic physicians and counsellors caring for their patients*.* Moreover, while the cinematic physician frequently fights the bureaucracy that impedes patients’ access to available drugs or novel treatment options, he or she is not an alienated or dull individual, but a dedicated and tireless hero struggling to help vulnerable patients. For example, while in *Extraordinary Measures*, dr. Stonehill is a hard-working and rational researcher whose revolutionary medical theories and innovative research help to develop an enzyme treatment for Pompe disease, dr. Fleming in *Midnight Sun* is pictured as a caring physician, who cares for a girl suffering from a life-threatening sensitivity to sunlight caused by a rare genetic disorder.

At the same time, even though the cinematic portrayals of RDs often do not reflect the current scientific knowledge, movies do take up some important topics in the field of RDs. Indeed, while biomedicine often focusses on the clinical aspects of RDs, movies highlight their ethical, psychosocial, legal or economic dimension, which are often overlooked in the scientific discourse. For example, while picturing experimental therapy for cystic fibrosis *Five Feet Apart* depicts patients’ experience with the disease, including coughing up blood, the inability to catch one’s breath, emotional distress related to anticipated death, patient’s dependence on the health system and the individual’s everyday struggle with hospital life. Similarly, both *Lorenzo’s Oil* and *Extraordinary Measures* show how deficits in scientific knowledge on RDs results in a confusing, chaotic, expensive and long-lasting diagnostic and therapeutic odyssey [58, 59]. They also illustrate how RD parents often become lay/self-experts on their child’s disease and the key players in the production of scientific knowledge. Moreover, although in both these movies the promise of a cure emerges, they also stress that RDs are too small to be easily funded and that finding a treatment is a time and money consuming enterprise. In *Children of the Dark* dr. Burnham while referring to the problem of orphan drugs explains to the parents of two girls suffering from xeroderma pigmentosum (XP) that it is not AIDS or cancer and therefore does not attract a lot of attention and that finding the cure for the RD may be ‘a billion dollars and generation away’. Thus, while movies often cover parents’ high hopes in research advances and their attempts to seek tests and drug trials, popular culture may help the public to understand how hard it is to handle the fiscal issues of RD drug development, how RD patients and their families struggle to get orphan drugs developed or how the bureaucracy impedes patients’ access to available drugs or novel treatment options. Simultaneously, movies like *Lorenzo’s Oil* or *Extraordinary Measures* stress the role of patient advocacy groups in RD research [60–62].

However, it is psychosocial issues related to RDs that are the most common tropes portrayed in the cinema. Indeed, all the analysed movies bring public attention to social stigmatization, isolation or discrimination resulting from patients’ rare genetic condition [63, 64]. Thus, while all the main characters in *The Elephant Man*, *Sixth Happiness*, *The Mighty*, *Simon Birch*, *Wonder* or *More Beautiful for Having Been Broken* experience prejudices, social exclusion and reduced life opportunities, in the movie *Mask* “Rocky”, who has craniodiaphyseal dysplasia, is also denied access to public school, and Jim’s daughters with XP in *Children of the Dark* refuse to organize their first sleepover with friends because ‘no one likes them’. Additionally, many movies emphasize that as a result of RDs the entire family faces stigmatisation, marginalization and discrimination from the neighbours, peers, work colleagues or local community.

Most movies also highlight how RDs affect patients’ entire life, influences one’s concept of self and become a source of self-stigma [65, 66]. For example, in *The Boy in the Plastic Bubble*, Tod, who suffers from severe combined immunodeficiency, becomes depressed after spending his entire life in incubator-like conditions, not being able to see the outside world and meet other people, and one day shouts loudly: “I’m so sick of it. I’m just feeling like a hospital case, like a weird kid who can’t even breath normally because I’ll get sick and die”. Similarly, the title character Alex, a girl with cystic fibrosis, confesses to her father: “This disease is getting much bigger than me… I have to do what it wants me to do… And maybe if I try to be its friend it would be so angry with me” (*Alex: The Life of a Child*). The Polish movie *Ondine* depicting the story of a young male suffering from congenital central hypoventilation syndrome who hides his disease from the girl he loves and who fears to reveal his condition to the outside world illustrates how disease invades every part of Cezary’s psychological self.

Another important theme depicted in movies includes the parents’ feelings of shock after receiving test results and their experience of self-blame [67, 68]. For example, both Michaela Odone in *Lorenzo’s Oil* and Jim in *Children of the Dark* expressed recurring feelings of guilt and a strong sense of responsibility for what had occurred to their children, and couldn’t stop blaming themselves for passing on “bad genes” to them. Finally, movies often stress how RDs affect family dynamics and relationships and creates tensions between spouses or between parents and their healthy children (*The Mighty*, *Paa*, *Wonder*, *Children of the Dark*).

## Conclusions

Although popular movies have a great educational potential [24, 27, 28], their utilization must be done under the premise that film is not science and that they are neither books nor scientific publications. Consequently, it should not surprise that rarely do the movies describe the clinical aspects of RDs as they mostly focus on patients’ problems of daily living. Moreover, the scientific elements that do appear on screen are often presented for the sake of a script rather than medical education. However, while the cinema serves as a unique ‘filter’ by which individuals perceive and experience RDs, it is also a tool that helps the moviemakers to shape the collective imagination. This is important because while interpreting the reality of RDs the public uses its own language that does not refer to numbers, scales or scientific diagrams and figures.

Thus, it should be also noted that the cinematic images of RDs are not isolated artefacts but unique products of a complex cultural activity: while they reflect, to some degree, the social imagination on such diseases, they also influence the public’s perception of RDs. Indeed, popular culture constitutes a unique symbolic resource and a “guide” which helps the audience to understand RDs. Even though the impact of popular culture on society is not decisive, it provides the public with images, examples and arguments for discussion on the clinical, psychosocial, ethical and economic implications of RDs. Cosnequently, as movies do co-create the interpretative context, they can significantly shape people’s cultural values and social attitudes towards such diseases and may affect changes in existing RD legislations.

To conclude, because popular culture is currently one of the most important mediums and resources from which the public derives its knowledge, it is hard to overestimate its influence on the public understanding and acceptance of RDs—the reason for this being that popular movies reach a much wider segment of society and a single cinematic picture may have a bigger impact on social attitudes towards RDs than formal education. This is especially so in that for individuals who do not have contact with or access to science, movies are often the only source of information on RDs. Consequently, while the need for stronger educational initiatives for both medical students and healthcare professionals is required, popular culture, including movies, can also serve as an educational tool in this field.

### Limitations

This study has some limitations. First, because the entire number of films picturing rare genetic diseases cannot be adequately determined, the selection of movies included into the analysis neither is nor could be representative. Second, only twenty popular movies on RDs were included into the analysis. Consequently, it would be desirable to extend the analysis and compare the content of popular films with different video formats that were not included, i.e. documentaries, short movies or even animations. Third, because the vast majority of the analysed movies were produced in the United States, the study sample is somehow biased as it under-represents European, Hispanic and Asian cinema. Fourth, as the entire analysis was performed by one author alone, there was a higher risk of subjectivity that might have influenced both the choice of the movies and the interpretation of the data. Finally, while it seems reasonable to argue that by using movies, popular culture puts the abstract and dire clinical information into the context of people’s lives and thus advocates for RDs and increases public awareness of such diseases, it is unclear if and how far the audience responds to and is influenced by movies. However, despite these limitations, some advantages of this study should also be acknowledged. Most importantly, as there is a scarcity of previous work on the topic, this research fills a gap in the literature regarding the cinematic representations of rare genetic diseases. Moreover, by providing new insights, it emphasises the role of popular movies in enhancing the public’s understanding of RDs.

## Data Availability

The datasets analyzed during the study are available from the corresponding author on reasonable request.

## Abbreviation

RD: Rare disease

## Footnotes

1 Filmweb, which is the largest Polish movie database, covers more than 667,000 movies [personal communication with the Marketing and Sales Manager], and IMDB provides information on more than 730,000 movies (both cinema and TV movies) (https://www.imdb.com/pressroom/stats/). Thus, in both cases the number of movies depicting RDs is very low.
